# Acute micro-outbreak of Chagas disease in the southeastern Amazon: a report of five cases

**DOI:** 10.1590/0037-8682-0687-2021

**Published:** 2022-08-22

**Authors:** Andreza Karoline Souza Barros de Brito, Débora Raysa Teixeira de Sousa, Edson Fidelis da Silva, Helton Jardys da Silva Ruiz, Ana Ruth Lima Arcanjo, Jessica Vanina Ortiz, Sabrina Silva de Brito, Denison Vital Jesus, Jorge Rubens Coelho de Lima, Kátia do Nascimento Couceiro, Mônica Regina Hosannah da Silva e Silva, João Marcos Bemfica Barbosa Ferreira, Jorge Augusto Oliveira Guerra, Maria das Graças Vale Barbosa Guerra

**Affiliations:** 1 Fundação de Medicina Tropical Dr. Heitor Vieira Dourado, Manaus, AM, Brasil.; 2 Universidade do Estado do Amazonas, Manaus, AM, Brasil.; 3 Hospital Maria da Gloria Dantas de Lima, Ipixuna, AM, Brasil.; 4 Fundação de Vigilância em Saúde Dra. Rosemary Costa Pinto, Manaus, AM, Brasil.; 5 Universidade Nilton Lins, Manaus, AM, Brasil.

**Keywords:** Chagas disease, Amazon, Epidemiology

## Abstract

**Background:**

Chagas disease is gaining importance in the Brazilian Amazon region as a differential diagnosis of febrile syndrome. The most recent microoutbreak occurred in Ipixuna, in Amazonas state.

**Methods:**

An epidemiological survey was conducted using parasitological and serological tests, and electrocardiographic analysis.

**Results:**

The patients belonged to one family and had ingested açaí acquired from Ipixuna. All patients reported fever and initially a thick blood smear test was done to identify *Trypanosoma cruzi*. Benznidazole treatment was administered to all patients.

**Conclusions:**

Knowledge of the epidemiological dynamics of Chagas disease allows us to improve control and management measures for this disease.

Acute Chagas disease (ACD) was first reported in 1980 in the Amazonas state^1^, and, since then, there have been reports of isolated cases of outbreaks^2^. The symptoms of the acute phase are non-specific, like fever, which is common in other endemic diseases of the Amazon region, such as malaria and arboviral infections^3^. Therefore, malaria microscopists have been trained to identify *Trypanosoma cruzi* through surveillance during the diagnosis of malaria^4^. 

Ipixuna municipality is located in the southwest of the Amazonas state, in the Juruá Region, 1,366 km from Manaus, (Figure 1 - Map produced by a contributor to the research group using QGIS 3.8 software). On March 14, 2021, 27 individuals including members of a single family and their close friends, consumed açaí collected and prepared by a single person on a farm located in the rural community known as Porto Rico. The house where the açaí was prepared has a palm leaf-thatched roof, and the kitchen is surrounded by palm trees, which are typical habitats for triatomines^5^. 

**FIGURE 1:**
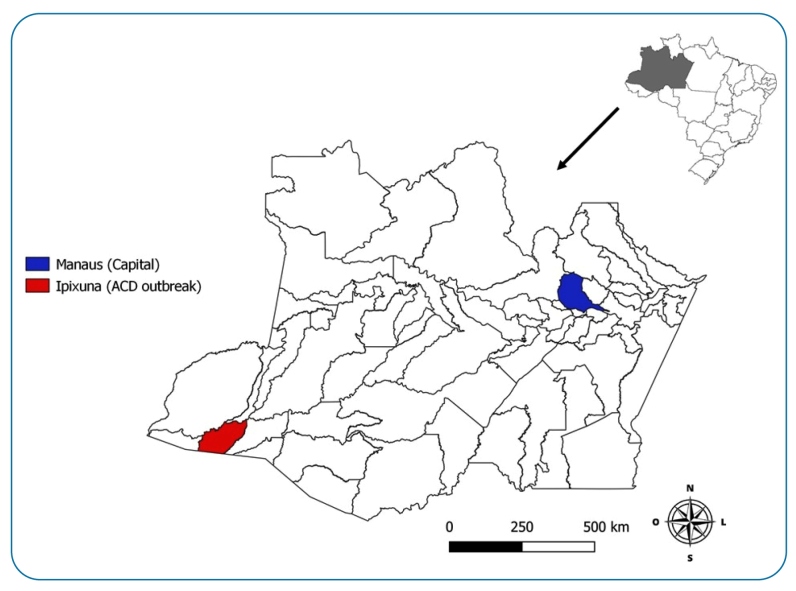
Map of Amazonas state, Brazil, showing the municipality of Ipixuna (in red) where the outbreak occurred and the municipality of Manaus (in blue), the state capital. Adapted by Jesus D (2021).

Between April 5 and 7, three cases of ACD were diagnosed in the town, resulting in a micro-outbreak. Professionals from the Fundação de Medicina Tropical Doutor Heitor Vieira Dourado (FMT-HVD), Fundação de Vigilância em Saúde do Amazonas Doutora Rosemary Costa Pinto (FVS-AM/RCP), and the Central Laboratory (LACEN) arrived at the town between April 10 and 16 to carry out the hematological and seroepidemiological survey, as well as for clinical care of the individuals exposed to contaminated açaí. The logistics for the investigations were difficult because it is a remote region, and access by river from the capital Manaus to Ipixuna usually takes 22 days. 

All exposed individuals, regardless of the presence of symptoms, underwent a thick blood smear (TBS) test for hemoparasite detection using the Walker method with Giemsa stain^6^. In addition, all blood samples collected were cultured on NNN medium (McNeal, Novy, Nicolle) for detection of parasites. The cultures were observed every week for up to 90 days^7^. Serological tests were also performed to detect anti-*T. cruzi* IgG antibodies using an indirect immunofluorescence assay (IFA; Bio-Manguinhos, Rio de Janeiro, RJ, Brazil; [≥90% sensitivity and specificity]), and an enzyme-linked immunosorbent assay (ELISA; Biolisa Chagas recombinante, Bioclin^®^, Belo Horizonte, MG, Brazil; [>99.9% sensitivity and 99.3% specificity]). For detection of anti-*T. cruzi* IgM antibodies, the samples were sent to a central laboratory (Fundação Ezequiel Dias, FUNED, MG, Brazil). Clinical examinations and standard 12-lead electrocardiogram (ECG; 10 mm/mV and 25 mm/s) were also performed. The Strout test was performed only for 22 patients who had a negative TBS test and, in all of them, the result was negative.

All participants provided written informed consent to participate in the study, and the study was approved by the Research Ethics Committee at the FMT-HVD, under approval number 33876120.2.0000.0005, in accordance with resolution 466/12 of the Brazilian National Health Council and the ethical guidelines of the 1975 Helsinki Declaration.

At the end of the investigations, five individuals had confirmed diagnosis of ACD (Table 1). The clinical and laboratory tests of the patients are described below.

**TABLE 1:** Laboratory tests of the five patients with positive thick blood smear test.

Patient	Thick blood	IgM (IFA)	IgG (IFA)	IgG (ELISA)	Blood culture
Case 1	Positive	R	R	R	Contaminated
Case 2	Positive	NR	R	R	Positive
Case 3	Positive	I	R	R	Positive
Case 4	Positive	R	R	R	Negative
Case 5	Positive	R	R	NR	Negative

**Key: R:** reactive; **I:** indeterminate (titer <1:40); **NR:** non-reactive; **IFA:** immunofluorescence assay; **ELISA:** Enzyme-linked immunosorbent assay.

**Case 1**: Male, 10 y; two days after ingesting açaí, he presented with fever, headache, abdominal pain, and vomiting, evolving in edema of the feet, hands, and face, in addition to persistent fever for 19 days. On April 5, 2021, his TBS test gave a positive result for *T. cruzi.* Titers of anti-*T. cruzi* with IFA were positive for IgM and IgG antibodies whereas, ELISA for anti-*T. cruzi* IgG antibody was negative. Blood culture results were compromised because of fungal contamination of the test samples. ECG showed no alterations. Benznidazole therapy was initiated immediately after diagnosis, and on the 20th day, he developed rashes on limbs indicating mild adverse reaction to the drug. Following first observed symptoms of adverse reactions to benznidazole, the medication was suspended for seven days, and corticosteroids (prednisone) were given. Subsequent re-introduction of benznidazole resulted in adverse effects again, such as skin rash and fever, and his general condition worsened. The medication was suspended again for ten days and, the patient was transferred to another health unit in another municipality in the neighboring state, where, under our guidance, benznidazole was reintroduced in reduced daily doses in combination with antiallergic drugs, thus completing the 60 days of treatment without further adverse effects. This patient’s family re-located to Cruzeiro do Sul in the state of Acre, where he was hospitalized and continued to be accompanied by professionals from the state of Acre.

**Case 2:** Male, 47 y; three days after ingesting açaí, the patient started presenting continuous fever, headache, back pain, and facial edema. On April 7, 2021, *T. cruzi* was identified in the TBS test. The patient tested negative for anti-*T. cruzi* IgM and positive for anti-*T. cruzi* IgG antibodies by both IFA and ELISA respectively, in addition to a positive blood culture. ECG showed signs of left atrial enlargement and non-specific alteration of ventricular repolarization in the inferior wall. He was put on benznidazole therapy on April 9, though he experienced mild epigastric pain as an adverse effect, with remission after taking omeprazole. The treatment lasted for sixty days without further complications.

**Case 3:** Male, 25 y; four days after ingesting açaí, the patient presented with fever, polyarthralgia, asthenia, and edema of the face and limbs. On April 7, 2021, TBS examination revealed *T. cruzi* infection. This patient showed indeterminate (1:20) titers of anti-*T. cruzi* IgM using IFA and positive anti-*T. cruzi* IgG titers by both, IFA and ELISA. His blood culture was positive, and ECG was normal. He was administered benznidazole on April 9 and had mild adverse reaction, described as worsening of facial edema after one day of therapy, which spontaneously regressed. The treatment was completed within sixty days without further complications.

**Case 4:** Female, 66 y; five days after açaí ingestion, the patient presented with fever, chills, headache, and edema of the face and limbs. She had reported earlier with dyspnea on exhaustion and worsening of it, even when at rest. The patient developed a generalized rash and itching. On April 3, she underwent an IgG/IgM rapid test for dengue with a reactive result. Her condition worsened and she required hospitalization and clinical support on April 12, 2021. She underwent TBS test during hospitalization, which revealed *T. cruzi* infection, confirming the diagnosis of ACD. Electrocardiography (ECG) showed an alteration in repolarization in the high lateral wall. The patient had positive titers for anti-*T. cruzi* IgM and anti- *T. cruzi* IgG by both, IFA and ELISA, but her blood cultures gave negative results. The patient was treated with benznidazole for eighty days because of her body weight (110 kg).

**Case 5:** Male, 25 y; two weeks after consuming açaí, he started to present with a continuous fever with chills, exanthema, polyarthralgia, retro-orbital pain, and, facial and hand edema. The patient used symptomatic medication, but the fever persisted. He underwent TBS examination on April 13, 2021, which revealed a *T cruzi* infection. The patient presented with positive anti-*T. cruzi* IgM and anti-*T. cruzi* IgG by IFA, and negative anti-*T. cruzi* IgG by ELISA. The blood culture was negative, and the ECG was normal. Treatment with benznidazole lasted for sixty days without complications*.*

In this report of five cases of ACD, involvement of family members was observed, as has occurred in other locations in the Amazon. These populations prepare their own food, often with inadequate hygiene; and consequently, food contaminated by *T. cruzi* gets consumed by the whole family^8^ ^,^ ^9^. An entomological investigation revealed presence of triatomines, *Rhodnius pictipes,* in palm trees close of the house where the açaí was prepared, one of which was found to be positive for *T. cruzi*.

In many ACD outbreaks that have occurred in the Amazon and surrounding regions, a varying percentage of individuals were found to have been exposed to the same food source but some of them did not develop symptoms associated with ACD and had negative results in diagnostic tests^10^.

ACD can be asymptomatic or marked by nonspecific symptoms. In our study, all reported patients had prolonged fever and four of the five patients had lower limb edema. None of the patients had severe manifestations, such as acute myocarditis or digestive hemorrhage^3^.

Regarding the screening tests for febrile syndrome, some patients underwent rapid tests for the diagnosis of dengue, which cannot differentiate between the presence of antibodies for the acute and chronic phases. Therefore, the positive results could indicate previous infections as the patients live in an endemic area. In addition, the dengue virus has high genetic homology with other flaviviruses, such as Zika and West Nile viruses, and with the chikungunya virus of the genus Alphavirus, and hence, cross-reactions and/or false-positive results can be observed in populations affected by such infections^11^ ^,^ ^12^. 

As the acute phase is characterized by high parasitemia, diagnostic methods for this phase consist of direct tests for identifying *T. cruzi* (direct fresh screening or concentration tests, such as Strout’s, microhematocrit, or buffy coat).^3^ In our study, the diagnosis of the index case occurred when *T. cruzi* was found in a TBS during the investigation of malaria, demonstrating the importance of training microscopists to identify to identify the different zoonoses present in the Amazon region. In ECG, alterations in ventricular repolarization were observed, which corroborate with other studies that report this alteration as the most common finding in the acute phase of Chagas disease^13^ ^-^ ^15^.

In Brazil, benznidazole is most frequently recommended for the treatment of ACD. Only the index case experienced severe adverse effects with this drug, one of which was non-bullous pruritic polymorphic erythema, which in severe cases may be accompanied by fever and lymphadenomegaly. Adverse reactions are an important cause of treatment interruption for ACD; however, the occurrence of such effects in children is not expected since they have greater tolerance to the drug^3^.

Reports of micro-outbreaks such as this one allows health managers and care workers to acquire knowledge of the local epidemiology to establish flowcharts for control measures, as well as conduct active searches for new cases, which should include the organization of serological surveys and, more importantly, case management in an early period of clinical evolution in order to achieve a serological cure. 
